# A high-resolution handheld millimeter-wave imaging system with phase error estimation and compensation

**DOI:** 10.1038/s44172-023-00156-2

**Published:** 2024-01-05

**Authors:** Yadong Li, Dongheng Zhang, Ruixu Geng, Zhi Lu, Zhi Wu, Yang Hu, Qibin Sun, Yan Chen

**Affiliations:** 1 School of Cyber Science and Technology, University of Science and Technology of China, Hefei, China; 2 School of Information Science and Technology, University of Science and Technology of China, Hefei, China

**Keywords:** Electrical and electronic engineering, Information technology

## Abstract

Despite the enormous potential of millimeter-wave (mmWave) imaging, the high cost of large-scale antenna arrays or stringent prerequisites of the synthetic aperture radar (SAR) principle impedes its widespread application. Here, we report a portable, affordable, and high-resolution 3D mmWave imaging system by overcoming the destructive motion error of handheld SAR imaging. This is achieved by revealing two important phenomenons: spatial asymmetry of motion errors in different directions, and local similarity of phase errors exhibited by different targets, based on which we formulate the challenging phase error estimation problem as a tractable point spread function optimization problem. Experiments demonstrate that our approach can recover high-fidelity 3D mmWave images from severely distorted signals and augment the aperture size by over 50 times. Since our system does not rely on costly massive antennas or bulky motion controllers, it can be applied for diverse applications including security inspection, autonomous driving, and medical monitoring.

## Abstract (web summary)

Millimeter-wave imaging using handheld devices suffers from significant degradation due to motion errors. Yan Chen and colleagues report an approach to correct the phase errors by introducing a reference target. The approach could recover high-fidelity 3D mmWave images from severely distorted signals.

## Introduction

Recent years have witnessed an increasing adoption of millimeter-wave (mmWave) imaging across various critical applications, including security scanning^1,2^, non-destructive testing^3,4^, and structural health monitoring^5–7^, among others. The impetus for this growth can be attributed to several advantages that render mmWave a more appealing imaging modality. In contrast to optical cameras, mmWave is more privacy-preserving, robust to illumination changes, and capable of penetrating various materials^8,9^. Furthermore, the non-ionizing nature of mmWave radiation allows for safer and broader usage compared to X-rays. To date, plenty of mmWave imaging systems have been deployed at major airports worldwide, where they play a pivotal role in detecting concealed threats. Additionally, promising breakthroughs have also showcased the ability of mmWave imaging to provide non-invasive diagnostic information pertaining to skin cancer^10,11^ and breast cancer^12,13^.

The fundamental capability that empowers various applications of mmWave imaging lies in its spatial resolution, which is intrinsically constrained by the aperture size (the number of antennas with a spacing of half of the wavelength)^14^. Existing high-resolution mmWave imaging systems are mainly built on the principles of either massive multiple-input multiple-output (MIMO)^15–20^ or synthetic aperture radar (SAR)^21^. With MIMO technology, a *N* × *N* antenna array can be formulated by 2*N* transceivers, thus achieving the desired spatial resolution with fewer antennas^22^. However, to produce high-resolution 3D mmWave images, hundreds of transceivers are still required, which greatly increases the hardware complexity and system cost. On the other hand, SAR synthesizes a virtual aperture by maneuvering the radar to transmit signals from different locations^23–25^. Nevertheless, bulky mechanical scanners or expensive tracking devices are indispensable to ensure that received signals from different locations can be combined coherently.

Recent advancements in mmWave radar technology have fostered the development of compact and affordable mmWave imaging systems by introducing SAR imaging to handheld settings^26^. However, the major challenge in handheld SAR imaging is that the fluidity of hand movements introduces non-linearity and non-uniformity in the virtual apertures, resulting in phase errors in received signals and severe distortion in the resultant images. Previous studies^26,27^ have resorted to expensive motion capture systems to achieve accurate device tracking during handheld scanning, which is not feasible for practical applications. To reduce system costs, some researchers have incorporated mobile localization methods for handheld mmWave SAR imaging. However, since the tracking precision fails to meet the stringent requirements (less than half a millimeter), they either necessitate an extended scanning time to accumulate recognizable images^28^ or time-consuming registration processes to obtain more precise positions^29^. Additionally, all aforementioned systems are based on time-domain imaging methods, which are suitable for non-linear motion but impose high computational burdens. To leverage efficient frequency-domain imaging techniques, which require linear and uniform sampling, several motion compensation methods^30–32^ have been proposed to compensate for the phase errors due to irregular scanning. Nonetheless, these approaches still depend on the premise of accurate device tracking, which is a non-trivial problem. In addition to signal processing-based methods, recent studies^29,33,34^ have directly input the amplitude image into deep neural networks to alleviate distortions caused by motion errors. However, they ignore the signal phase critical for phase error compensation and simplify it as an image super-resolution problem. Moreover, these approaches require substantial training data to ensure the robustness of the deep learning models, leading to increased deployment costs. Consequently, a low-cost and efficient phase error correction solution is essential for practical handheld SAR imaging systems.

Here, we report a handheld mmWave imaging system that combines the advantages of MIMO technology and SAR principle, thus augmenting the aperture size of commercial-off-the-shelf mmWave devices by over 50x on average. Different from conventional approaches that focus on obtaining more precise device locations, our objective is to acquire the optimal point spread function (PSF) of the handheld synthetic array to effectively combat motion errors. Specifically, we first make a deep investigation into the root cause of phase errors, uncovering its spatial asymmetry which means that motion errors along different directions exert different influences on image quality. Moreover, we observe that the phase errors of different targets exhibit a local similarity, making it possible to approximate the phase error of the imaging target with another reference target. Based on these findings, we focus on the direction most susceptible to motion errors and estimate the optimal PSF based on the quadratic feature of the ideal phase history. Once obtaining the estimated PSF, we can derive and compensate for the phase errors caused by manual scanning and reconstruct the target with the efficient frequency-domain imaging method. Extensive experimental validations demonstrate the efficacy of our proposed imaging system in restoring targets from heavily distorted initial measurements, showcasing remarkable enhancements in both Peak Signal-to-Noise Ratio (PSNR, 4.54 dB) and Structure Similarity Index Measure (SSIM, 31.19%). As a result, our study can be deployed in mmWave applications that necessitate high mobility, cost-effectiveness as well as high-resolution imaging.

## Methods

### Spatial asymmetry of phase errors

**Fig. 1:**
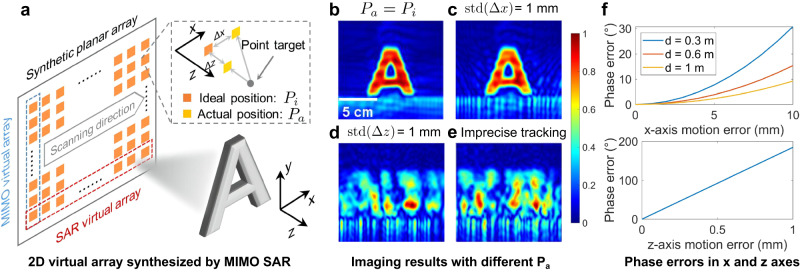
The spatial asymmetry of phase errors. **a** An ideal planar aperture can be synthesized by mechanically moving a linear multi-input-multi-output (MIMO) array. In handheld synthetic aperture radar (SAR) imaging, the actual positions of the antenna will deviate from the ideal positions. In the established Cartesian coordinate system, the x-axis, y-axis, and z-axis denote the horizontal scanning, vertical, and depth directions, respectively. Δ*x* and Δ*z* denote the motion error along the x-axis and z-axis, respectively. **b** The imaging result obtained using time-domain back-projection^53^ with the ideal antenna positions, which appears clear and well-defined (see the experimental setup in Supplementary Note 1). **c**, **d** These demonstrate the imaging results when statistical position errors with a standard deviation (std) of 1 mm (see Supplementary Fig. 1a, b) are added to the x-axis and z-axis, respectively. The z-axis position errors introduce the image’s most significant distortions and blurring. **e** The blurred image is generated by the recorded positions of a tracking camera (see Supplementary Fig. 1c), showcasing the impact of imprecise localization. **f** The z-axis motion errors result in much larger phase errors than the x-axis and y-axis motion errors, where *d* denotes the z-axis distance between the target and the antenna. Overall, these results highlight the importance of accurate position estimation in handheld SAR imaging, particularly in mitigating z-axis motion errors due to the asymmetry of phase errors along different directions.

To achieve 3D mmWave imaging, a 2D planar aperture is required to obtain angle resolution along the azimuth and elevation directions. Combining MIMO technology with SAR principle makes it possible to synthesize a virtual planar array more efficiently. Figure 1a illustrates the synthesis of an ideal planar aperture by horizontally moving a linear MIMO array using a mechanical scanner. After a multistatic-to-monostatic transformation^35^, the instantaneous position of one arbitrary antenna can be denoted as $({x}^{{\prime} },{y}^{{\prime} },0)$ and a single point on the imaging target can be represented by (*x*, *y*, *z*) with a reflectivity function *σ*(*x*, *y*, *z*). Under the Born approximation for the scattering field and an isotropic antenna assumption^36^, the received signal can be expressed as:

1 $$s({x}^{{\prime} },{y}^{{\prime} },0)=\sigma (x,y,z){{{{{{{{\rm{e}}}}}}}}}^{-{{{{{{{\rm{j}}}}}}}}2kR},$$

where $k=\frac{2\pi f}{c}$, and *f*, *c*, and *k* represent the frequency, speed of propagation, and wavenumber, respectively. *R* is the slant range between the antenna and the imaging points, which can be denoted as:

2 $$R=\sqrt{{(x-{x}^{{\prime} })}^{2}+{(y-{y}^{{\prime} })}^{2}+{z}^{2}}.$$

Now let’s consider the handheld SAR imaging scenario where the motion trajectory of the antenna deviates from the ideal trajectory. In this case, the actual position of the antenna is given by $({x}^{{\prime} }+{{\Delta }}x,{y}^{{\prime} }+{{\Delta }}y,{{\Delta }}z)$, and the ideal instantaneous range *R* changes to *R*_e_ as:

3 $${R}_{{{{{{{{\rm{e}}}}}}}}}=\sqrt{{(x-{x}^{{\prime} }-{{\Delta }}x)}^{2}+{(y-{y}^{{\prime} }-{{\Delta }}y)}^{2}+{(z-{{\Delta }}z)}^{2}}$$

As a result, the received signal with motion error becomes:

4 $$s({x}^{{\prime} }+{{\Delta }}x,{y}^{{\prime} }+{{\Delta }}y,{{\Delta }}z)=\sigma (x,y,z){{{{{{{{\rm{e}}}}}}}}}^{-{{{{{{{\rm{j}}}}}}}}2k{R}_{{{{{{{{\rm{e}}}}}}}}}}.$$

Analyzing the influence of motion errors on different axes reveals that the resultant phase error distribution exhibits a spatial asymmetry. Specifically, Fig. 1b shows the ideal imaging result when there is no motion error. However, if we add the same motion error along different axes, we can find that the z-axis motion errors have a considerably more significant impact than the x and y-axis errors, as demonstrated in Fig. 1c, d. This is because SAR imaging is highly sensitive to localization errors along the z-axis due to the resultant significant phase errors. For example, consider a target located 1 m away from a 77 GHz mmWave device, as depicted in Fig. 1a, a 1 mm shift in the x or y-axis would only introduce a signal propagation distance change of 0.0005 mm, resulting in a phase error of Δ*Φ*_*x*/*y*_ = 0.092°. However, a 1 mm deviation in the z-axis would lead to a phase error of Δ*Φ*_*z*_ = 184.8°, which is significantly larger and has a considerable impact on image quality, as demonstrated in Fig. 1f.

This observation makes it possible to relieve the burden of motion compensation in 3D space and focus only on the dominant direction (i.e., only considering the motion error in the z-axis Δ*z* and approximating Δ*x* and Δ*y* as 0). Moreover, in near-field SAR imaging settings, *R*_e_−*R* can be well approximated by Δ*z*^37^. Hence, if the actual positions of the handheld device can be precisely localized, the signal with a z-axis motion error Δ*z* can be closely mapped to the ideal one by compensating for the phase error as^37^:

5 $$s({x}^{{\prime} },{y}^{{\prime} },0)\approx s({x}^{{\prime} },{y}^{{\prime} },{{\Delta }}z){{{{{{{{\rm{e}}}}}}}}}^{{{{{{{{\rm{j}}}}}}}}2k{{\Delta }}z}.$$

Existing mobile localization methods, however, fall short of the required tracking accuracy. The impact of localization errors can be observed in Fig. 1e, where the imaging result generated with the imprecise localization shows a severely defocused and distorted image compared to the ground-truth SAR image. The reflections of the target are spread throughout the entire image area, and the target’s shape is unrecognizable. Hence, the key to successful handheld SAR imaging lies in accurately estimating and compensating for phase errors caused by deviations in the z-axis.

### Local similarity of phase errors

Estimating z-axis phase error, however, is challenging for two reasons. First, the received signal phase is a superimposition of all scatters in the imaging scene, including the imaging target and other uninterested objects, which have different impacts on the phase of the final received signal. Hence, it is difficult to directly estimate phase errors from such a combination of diverse phase error components. Second, the distribution of motion errors caused by handheld scanning is complicated and unclear. Consequently, specific assumptions about phase error distribution which could reduce the difficulty of phase error estimation are also impractical.

**Fig. 2:**
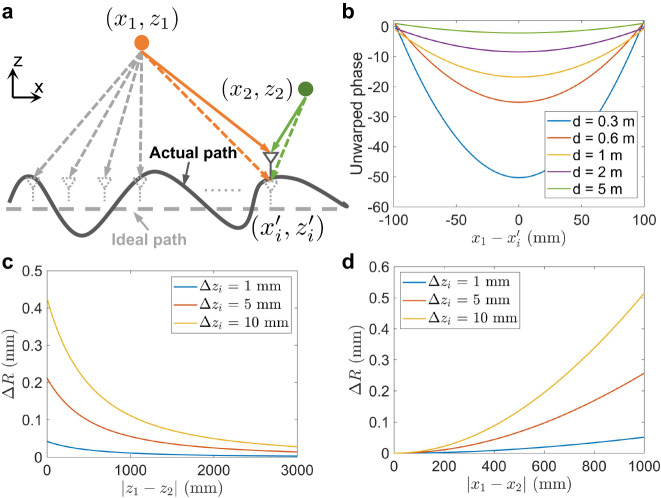
The local similarity of phase errors. **a** The changes in propagation distances of the ideal array and the impact of z-axis motion error on different point targets. (*x*_1_, *z*_1_), (*x*_2_, *z*_2_), and $({x}_{i}^{{\prime} },{z}_{i}^{{\prime} })$ represent the coordinate of two point targets and *i*th ideal antenna, respectively. **b** The simulated phase histories of point target (*x*_1_, *z*_1_) with different *d*, where $d={z}_{1}-{z}_{i}^{{\prime} }$. For uniform and linear virtual arrays, the phase histories follow quadratic curves, and the curvature decreases as the distance between the antenna and the target increases. **c** illustrates the distribution of Δ*R* (the difference in the round-trip propagation error between these two targets) with ∣*z*_1_ − *z*_2_∣, assuming *x*_2_ = 300 mm, $({x}_{i}^{{\prime} },{z}_{i}^{{\prime} })=(0,0)$, and (*x*_1_, *z*_1_) = (0, 1000) mm. Δ*z*_*i*_ is the motion error along the z-axis. **d** illustrates the distribution of Δ*R* with ∣*x*_1_ − *x*_2_∣, assuming *z*_2_ = 3000 mm, $({x}_{i}^{{\prime} },{z}_{i}^{{\prime} })=(0,0)$, and (*x*_1_, *z*_1_) = (0, 300) mm. It reveals that the phase errors of two targets can exhibit considerable similarity even if they are far from each other.

To tackle these challenges, we propose combating the z-axis phase errors by leveraging the characteristic of ideal signal phase variations and the local similarity exhibited by different targets’ phase errors. We start with the analysis of the ideal phase history of an isolated point target. Suppose the radar moves along a linear path with a constant velocity *v*, as illustrated in Fig. 2a, and there is a single point target (the orange point) in front of the radar with a position of (*x*_1_, *z*_1_). For the *i*^*th*^ transmitting location, the received signal of this point target can be expressed as:

6 $$s({x}_{i}^{{\prime} },{z}_{i}^{{\prime} })=\sigma ({x}_{1},{z}_{1}){{{{{{{{\rm{e}}}}}}}}}^{-{{{{{{{\rm{j}}}}}}}}2k\sqrt{{({x}_{1}-{x}_{i}^{{\prime} })}^{2}+{({z}_{1}-{z}_{i}^{{\prime} })}^{2}}},$$

where *σ*(*x*_1_, *z*_1_) is the reflection coefficient of target located at (*x*_1_, *z*_1_). $({x}_{i}^{{\prime} },{z}_{i}^{{\prime} })$ is the ideal position of *i*th transmitting antenna. Hence, the unwrapped phase history *H* of the point target can be represented as:

7 $$H={\left\{U\left(2k\sqrt{{({x}_{1}-{x}_{i}^{{\prime} })}^{2}+{({z}_{1}-{z}_{i}^{{\prime} })}^{2}}\right)\right\}}_{i = 1}^{n},$$

where *n* is the number of total transmitting locations. *U* denotes the phase unwrap function. From Eq. (7), we observe that under near-field conditions, when the radar moves linearly and uniformly (*x*_*i*_ increases linearly while *z*_*i*_ remains unchanged), *H* follows a quadratic curve, as illustrated in Fig. 2b. This fundamental characteristic, actually, can be utilized as a strong prior to estimate the phase errors of the point target by measuring its difference with the ideal phase history.

However, obtaining the phase errors of a specific point target does not necessarily solve our problem because targets at different locations usually exhibit different reflected properties and phase variations. To exploit the correlation of phase errors between different targets, let’s consider two distinct point targets in the imaging scene located at (*x*_1_, *z*_1_) and (*x*_2_, *z*_2_), respectively, as depicted in Fig. 2a. For a single virtual antenna, the ideal antenna position is $({x}_{i}^{{\prime} },{z}_{i}^{{\prime} })$, while the actual antenna position contains a z-axis motion error of Δ*z*_*i*_ and is given by $({x}_{i}^{{\prime} },{z}_{i}^{{\prime} }+{{\Delta }}{z}_{i})$. Hence, we can represent the difference in the round-trip propagation error between these two targets as follows:

8 $$\Delta {R}_{1}	 = \, \sqrt{{({x}_{1}-{x}_{i}^{{\prime} })}^{2}+{({z}_{1}-{z}_{i}^{{\prime} })}^{2}}\\ 	 -\sqrt{{({x}_{1}-{x}_{i}^{{\prime} })}^{2}+{({z}_{1}-{z}_{i}^{{\prime} }-\Delta {z}_{i})}^{2}},$$

9 $$\Delta {R}_{2}	 = \, \sqrt{{({x}_{2}-{x}_{i}^{{\prime} })}^{2}+{({z}_{2}-{z}_{i}^{{\prime} })}^{2}}\\ 	 - \sqrt{{({x}_{2}-{x}_{i}^{{\prime} })}^{2}+{({z}_{2}-{z}_{i}^{{\prime} }-\Delta {z}_{i})}^{2}},$$

10 $${{\Delta }}R=| (| {{\Delta }}{R}_{1}| -| {{\Delta }}{R}_{2}| )| ,$$

where Δ*R*_1_ and Δ*R*_2_ denote the round-trip propagation error of target (*x*_1_, *z*_1_) and target (*x*_2_, *z*_2_), respectively. Δ*R* represents the difference between ∣Δ*R*_1_∣ and ∣Δ*R*_2_∣. Obviously, the smaller the Δ*R* is, the closer these two targets’ phase errors will be. Figure 2c, d illustrates the spatial distribution of Δ*R* with ∣*z*_1_ − *z*_2_∣ and ∣*x*_1_ − *x*_2_∣, respectively. We can observe that it is easy to find a region that minimizes Δ*R*, referred to as the local similarity of phase errors of different targets. Therefore, by setting a point target according to the distribution of Δ*R*, its estimated phase error can be quite close to the phase error of the imaging target.

### Estimation of phase errors

**Fig. 3:**
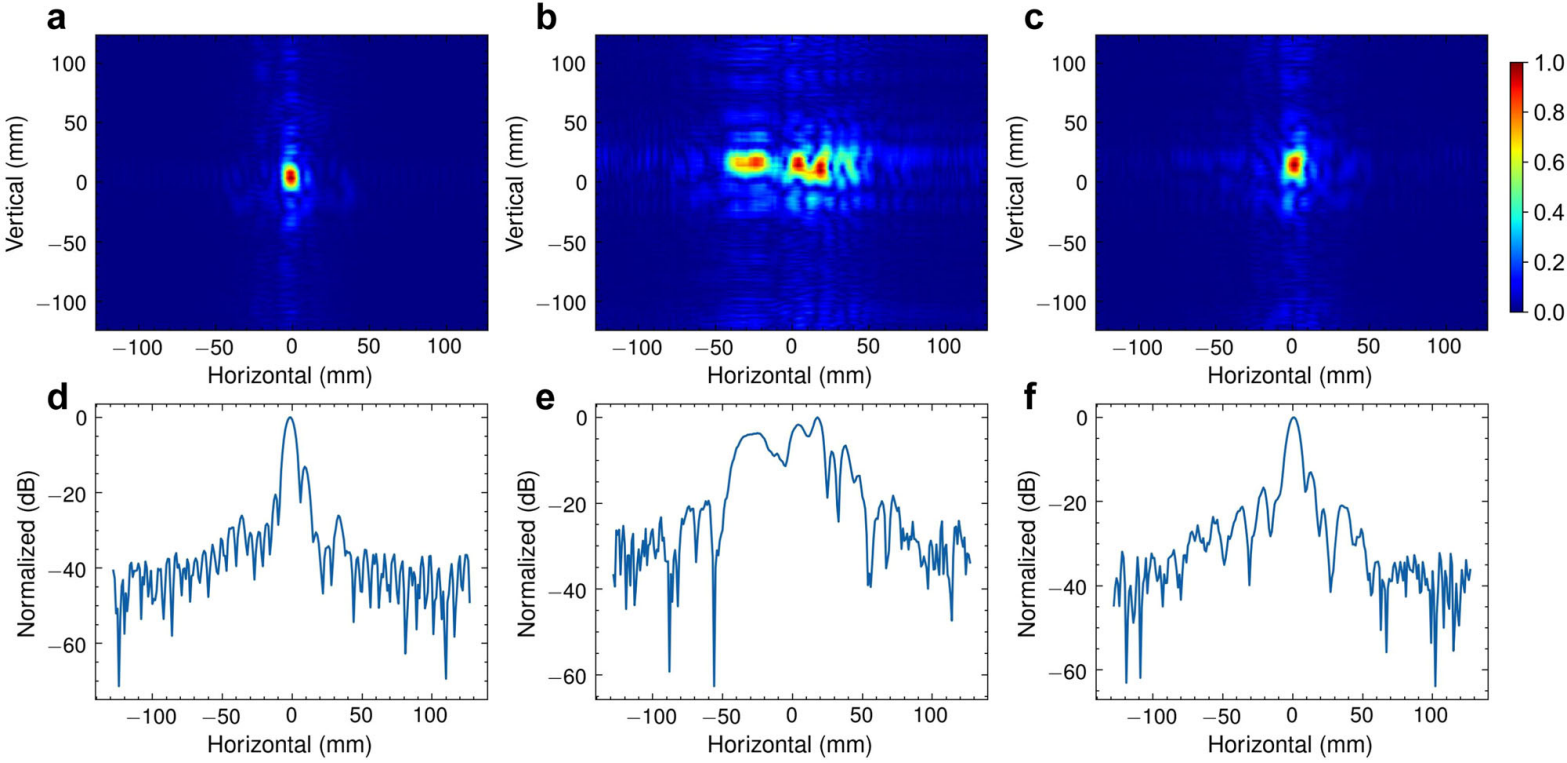
The optimization results of 1D and 2D point spread function (PSF) (see the experimental setup in Supplementary Note 1–3). **a** represents the 2D PSF obtained from mechanical scanning, while (**d**) displays the corresponding 1D PSF. Notably, the PSFs of handheld scanning, depicted in (**b**) and (**e**) for 2D and 1D, respectively, suffer from distortion and blurring caused by motion errors (see Supplementary Fig. 2). However, the proposed optimization technique successfully mitigates the impact of motion errors on the PSFs. **c**, **f** These demonstrate the optimized 2D and 1D PSFs of handheld scanning, respectively. These optimized PSFs effectively combat the distortions induced by motion errors, leading to substantially improved image quality.

PSF, the impulse response of an ideal point target, is usually utilized to characterize the capability of an imaging system. For SAR systems, the 1D PSF of a linear array or 2D PSF of a planar array reflects the angle resolution and focusing ability of the antenna array, and higher-quality PSFs correspond to improved imaging performance. In the context of this study, Fig. 3a–e visually demonstrates the notable contrast between the PSFs derived from mechanical scanning and the PSFs obtained through handheld scanning. Evidently, the PSFs resulting from handheld scanning experience considerable distortion due to motion errors.

To tackle the motion errors caused by manual scanning, we propose to optimize and restore a high-fidelity PSF to obtain a reliable estimation of phase errors. This is achieved based on previous findings, namely: (i) when the radar moves linearly and uniformly, the phase history of a single point target follows a quadratic curve, and (ii) in the *x*-*z* plane, targets located at different distances can exhibit similar phase errors. Accordingly, we first manually designate a point target within the imaging scene as a reference, ensuring both reference and imaging targets are observed by the radar. Subsequently, we attempt to obtain the optimized PSF by fitting the phase history of this point target with the corresponding ideal quadratic phase history. Through this process, we can measure the phase error of this point target by quantifying the discrepancy between its actual and ideal phase history. Finally, the estimation can be utilized to compensate for the phase history of the imaging target and generate the corrected SAR image. Figure 3c, f shows the PSF optimization results, which appear clear and well-focused (see the estimated deviation from a regular acquisition and phase error in Supplementary Fig. 2). It is noteworthy that our approach requires neither expensive tracking devices, nor iterative optimization processes, which reduces the system cost and imaging time. This enables a more affordable and efficient handheld SAR imaging system, which can be highly beneficial in various applications, such as security checks, SLAM, and industrial inspections.

### System architecture

**Fig. 4:**
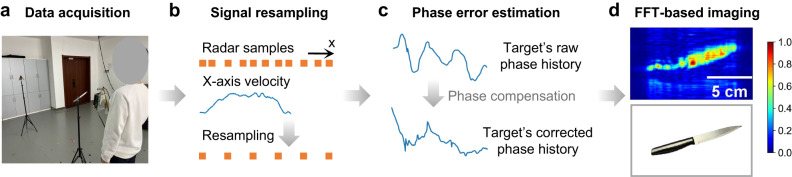
The framework of the handheld mmWave imaging system. **a** A multi-input-multi-output radar and a co-located tracking camera are used to collect radar signals and device trajectory with manual scanning. **b** The collected radar frames are resampled to uniform spacing along the x-axis using the velocity recorded by the camera. **c** The point spread function optimization is subsequently employed to estimate and compensate for the target’s phase history. **d** Images are generated with the Fast Fourier Transform (FFT)-based synthetic aperture radar imaging method.

Based on the above discussions, we build our handheld imaging system with three key components: signal resampling, PSF optimization, and SAR imaging, as depicted in Fig. 4. Specifically, Fig. 4a illustrates the data collection setup. The operational flow begins with resampling the collected signals to uniform spacing to apply the efficient FFT-based imaging methods, as shown in Fig. 4b. This is achieved by oversampling the radar frames and tracking the radar’s motion using a cost-effective visual tracking device. Subsequently, to address the non-linearity along the z-axis, we employ the previously described PSF optimization and obtain the compensated SAR echoes, as shown in Fig. 4c. Finally, the Range Migration Algorithm (RMA)^38,39^, a fast frequency-domain near-field imaging method, is utilized to reconstruct the SAR image, as depicted in Fig. 4d. Figure 4a also highlights its potential for application in security checks, where handheld scanning and high-quality imaging are essential. Traditional approaches rely on metal detectors which can only detect the presence of metal objects while we can reconstruct the exact shapes of hidden objects, enabling accurate identification of potential threats.

As depicted in Fig. 4a and Supplementary Fig. 6, we prototype our imaging system with the TI four-chip cascaded mmWave radar operating within the frequency range of 76-81 GHz. With the MIMO technique, the radar can formulate a virtual linear array consisting of 86 elements. This arrangement enables the creation of a 2D planar array by moving the radar along the orthogonal direction of its linear array dimension. As a result, the system can reconstruct specific objects through a single scanning process, thereby reducing the scanning time and improving users’ experience. The radar is co-located with the Intel RealSense T265 tracking camera, which captures the radar’s movement along the x-axis and is utilized to resample the collected data to uniform spacing. Since our focus lies solely on recording the x-axis movement, which exhibits a higher tolerance for tracking errors, alternative cost-effective mobile localization methods may also be suitable.

### Implementation details

#### PSF optimization

Suppose a monostatic antenna moves in a handheld configuration and transceives Frequency Modulated Continuous Wave (FMCW) signals. The imaging object and the reference target are situated at different distances from the center of the antenna’s trajectory. By leveraging the principles of FMCW, we can separate the two targets in the range domain by performing FFT (range-FFT) along each reflected pulse, assuming the distance between them is larger than the device’s range resolution. Based on the previous discussion that the phase history of a point target for an ideal trajectory follows a quadratic curve, we can proceed to estimate the ideal phase variation of the reference point target by optimizing the following function:

11 $${\hat{\phi}}_i = \mathop{{{{{\rm{arg}}}}} \, {{{{{\rm{min}}}}}}}\limits_{\alpha, \beta, \gamma} \mathop{\sum}\limits_{i=1}^n (\alpha i^2 + \beta i + \gamma - \phi_i)^2,$$

where $\hat{{\phi }_{i}}$ represents the estimated ideal phase variation of the reference point target from the received signal at *i*th location. *ϕ*_*i*_ is the corresponding disturbed phase variation extracted with range-FFT. *n* is the number of received pulses. *α*, *β,* and *γ* are the coefficients of the target curve. This function finds the optimal *α*, *β,* and *γ* values which can minimize the difference between the actual phase variations of the reference and its ideal phase variations. Once the estimated ideal phase history has been obtained, the phase error induced by handheld motion can be compensated as:

12 $$\hat{{s}_{i}}={s}_{i}{{{{{{{{\rm{e}}}}}}}}}^{{{{{{{{\rm{j}}}}}}}}(\hat{{\phi }_{i}}-{\phi }_{i})},$$

where $\hat{{s}_{i}}$ and *s*_*i*_ denote the compensated and the motion error corrupted *i*th received signal, respectively.

Notably, in this paper, a linear array is employed to facilitate MIMO SAR imaging. Hence, at each time step *i*, there will be multiple phase variations corresponding to multiple antennas. Although it is possible to estimate the phase error for each antenna, such an approach would increase computational costs and exacerbate the impact of incorrect estimations. To address this issue, we make the reasonable assumption that the primary phase error arises from translational motion error, considering that the device only needs to be moved along a single direction for a distance of approximately 20 cm to 30 cm. As a result, the antennas at the same timestep exhibit similar phase errors, which can be obtained by selecting the phase history of a single antenna to optimize in Eq. (11). Moreover, in the experiments, we find that if the x-axis position of the reference point target deviates from the x-axis center of the scanning trajectory, setting *α* as 0 would lead to better performance due to the incomplete quadratic feature of the ideal phase history.

#### Hardware configuration

Our imaging system is implemented with the TI four-chip cascaded mmWave radar, which consists of 12 transmitting antennas and 16 receiving antennas. We activate all the transceivers to achieve an 86-element virtual linear array. To collect the reflected signals in real-time, we attach a data capture board to the radar, which can transfer the collected data to a laptop through an Ethernet cable. The mmWave radar transmits FMCW signals which have the following parameters: start frequency, 77 GHz; ADC sampling rate, 8 Msps; chirp slope, 38.5 MHz/*μ*s; chirp duration, 40 μs; the number of ADC samples, 256; frame periodicity, 10 ms for manual scanning and 50 ms for mechanical scanning, respectively. The mmWave radar is co-located with the Intel RealSense T265 camera, which has two fish eye cameras and one inertial motion unit to track the device’s motion. Since the camera’s pose updating rate (approximately 30 Hz) is lower than the radar’s frame rate, we employ cubic interpolation on the trajectory obtained by the camera, to get the radar’s position for each transmitted frame.

#### Scanning settings

The mechanical scanning is achieved by moving the MIMO radar over a distance of 200 mm along the x-axis with a motion controller. The moving speed is 20 mm/s, resulting in 200 sampling pulses with a spacing of 1 mm.

The users are instructed to hold and move the MIMO radar over a distance of ~20 cm along the x-axis (see the experimental setup in Supplementary Fig. 6). The radar’s frame rate is set at 10 ms, while the user’s movement speed is restricted to no more than 0.2 m/s, in order to satisfy the Nyquist-Shannon sampling theorem. It is worth noting that by increasing the frame rate, we can achieve higher scanning speeds; however, this comes at the expense of heavier data processing.

## Results

### Aperture size augmentation

**Fig. 5:**
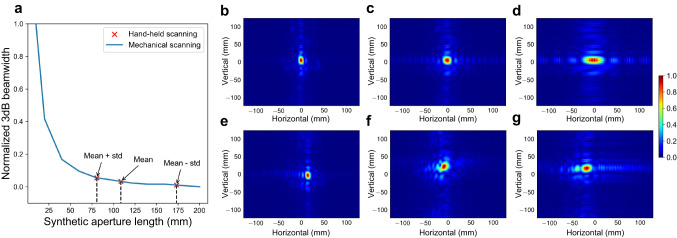
Effect of aperture size augmentation by comparing the point spread function (PSF) between mechanical and handheld scanning (see the experimental setup in Supplementary Note 1–3). **a** The blue line shows the 3 dB beamwidth for different aperture lengths of mechanical scanning normalized to the value when the aperture length is 10 mm. The red markers show the average (plus/minus a standard deviation (std)) 3 dB beamwidth of the PSFs associated with the 100 manual scanning of 20 cm normalized to the same value as the blue line. **b**, **c**, **d** These depict the 2D PSFs obtained from different aperture lengths of 200 mm, 100 mm, and 40 mm, respectively. **e**, **f**, and **g** These illustrate the 2D PSFs resulting from handheld scanning. These handheld PSFs are specifically chosen to match the 3 dB beamwidth values equivalent to the point of mean-std, mean, and mean+std in (**a**). Considering that half of the wavelength of the mmWave device is 1.9 mm and the interval of two adjacent pluses during mechanical scanning is 1 mm, we can draw the conclusion that our approach can achieve imaging performance equivalent to a 50-antenna linear array with only one antenna. In other words, the mmWave device’s aperture size is augmented by 50 times on average with our method.

To evaluate the ability to augment the aperture size of our handheld imaging system, we employ the 3 dB beamwidth of the 1D PSF as a measure and compare it to different lengths of apertures generated by mechanical scanning. In this evaluation, we position two corner reflectors in front of the imaging device: one serves as the imaging target, located at a range of 0.65 m, and the other as the reference target, positioned at a range of 1.8 m. A total of 100 manual scanning samples are collected from two different users, with each user having 50 samples (see how we calculate the estimated deviation from a regular acquisition in Supplementary Note 5 and the histogram of estimated deviation in Supplementary Fig. 4a). While the length of each manual scanning is ~200 mm, it does not necessarily mean that a 200 mm handheld scanning aperture is directly comparable to a 200 mm mechanical scanning aperture. This is because even after applying the proposed phase error compensation approach, residual phase errors may still be present. To determine the equivalent mechanical scanning imaging performance, we compute the normalized 3 dB beamwidth for different aperture lengths of mechanical scanning, as illustrated in the blue line of Fig. 5. Then we mark the average (plus/minus a standard deviation) 3 dB beamwidth of the PSFs associated with the 100 manual scanning samples as red to evaluate the imaging performance of handheld scanning. As a result, Fig. 5a shows that the average and best imaging performance of 200 mm handheld scanning is approximately equivalent to 100 mm and 175 mm mechanical scanning, respectively. Moreover, Fig. 5b–g depicts the 2D PSFs obtained from mechanical scanning and handheld scanning, respectively, which demonstrates that the proposed approach can effectively combat motion errors.

### Comparison with baseline

**Fig. 6:**
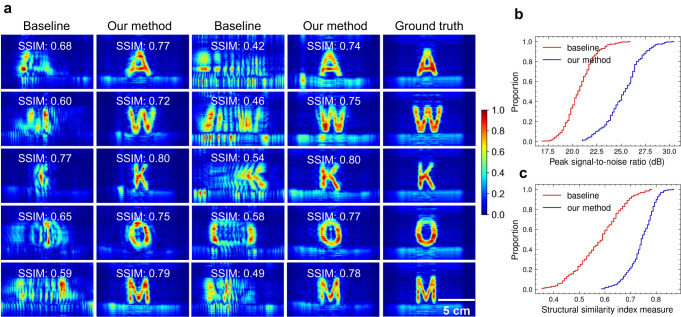
Comparison with baseline^32^ (see the experimental setup in Supplementary Note 1–3). **a** Qualitative reconstruction results of different structural similarity index measure (SSIM) improvements (see more qualitative results in Supplementary Fig. 3). The empirical cumulative distribution functions of peak signal-to-noise ratio (**b**) and SSIM (**c**) are employed to quantitatively evaluate the resulting images, which demonstrate that our method brings a noteworthy average enhancement of 4.54 dB in peak signal-to-noise ratio and a substantial 31.19% improvement in SSIM.

To evaluate the imaging performance of our system, we collect 200 samples from four different users imaging ten different metal letters, with each user having 50 samples (see the histogram of estimated deviation from a regular acquisition in Supplementary Fig. 4(b)). To demonstrate the superiority of our approach, we make a comparison with the method in ref. ^32^. It is noteworthy that the core of ref. ^32^ lies in compensating the squiggle path to the uniform path in the x-y plane and assuming there is no z-axis motion error, which essentially functions the same as the signal resampling in our system. As demonstrated in Fig. 6a, even when compensating for the non-uniform motion error using the camera, the resulting SAR images still exhibit significant distortion due to z-axis motion error. Conversely, by mitigating phase errors through PSF optimization, the targets can be well-focused with high fidelity. The ECDF of PSNR and SSIM, presented in Fig. 6b, respectively, reveal a noteworthy improvement in image quality after applying phase correction. Furthermore, our PSF optimization does not necessitate iterative or computationally intensive processes. As a result, the average time required to estimate and compensate for the phase errors of a single image is approximately 60 ms using an Intel Core i7-11700K CPU (see Supplementary Note 4), demonstrating its superior efficiency and potential for real-time applications.

### Different reference positions

**Fig. 7:**
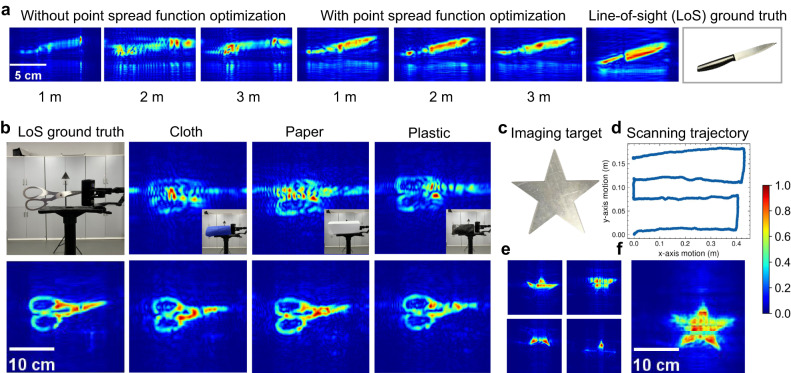
Experimental results in different scenarios (see the experimental setup in Supplementary Note 1–3). **a** The reconstruction results for various reference positions. In this experiment, a knife serves as the imaging object and is positioned at a distance of 0.3 m from the virtual planar array. Meanwhile, the reference is chosen at 1 m, 2 m, and 3 m, respectively. **b** Non-line-of-sight imaging results for diverse occlusions. In this context, a scissor acts as the imaging object, positioned 0.35 m away from the virtual planar array. The reference is chosen at a distance of 2 m. To simulate non-line-of-sight scenarios, three distinct materials—cloth, paper, and plastic—are employed to cover the scissors. The first row represents the imaging result after signal resampling, while the second row depicts the imaging result achieved through point spread function optimization. **c**–**f** These show the multi-scanning reconstruction results. **c** Optical image of the target, with a width of 25 cm and a height of 25 cm. **d** The handheld scanning trajectory is along the *x* and *y* axes, showcasing the path of data collection. **e** The imaging results for each individual scanning after point spread function optimization. **f** The fused image generated from multiple scanning with point spread function optimization.

As described in Fig. 2c, it is straightforward to find a position for the reference to make the phase error of the two objects fairly close. Moreover, the difference in z-axis positions between the imaging object and the reference object does not significantly impact the imaging performance. Figure 7a exhibits the reconstruction results for various reference positions, demonstrating that the imaging object in each scenario can be successfully refocused (see quantitative analysis in Supplementary Fig. 5). Hence, our approach can be flexible and easy to be employed in practical scenarios.

### Non-line-of-sight imaging

Compared with optical imaging, mmWave can penetrate diverse non-metallic materials, making it suitable for Non-Line-of-Sight (NLoS) imaging to detect hidden objects. To illustrate the see-through ability of our imaging system, we cover the imaging object with various materials (i.e., paper, plastic, and cloth), then perform handheld scanning to reconstruct the concealed objects. Figure 7b illustrates the successful reconstruction of hidden objects using our handheld imaging system, despite the presence of different types of occlusions. This outcome demonstrates the system’s potential in non-destructive testing and security inspections, where it can facilitate the detection of concealed items.

### Imaging with multi-scanning

Previous experiments have successfully demonstrated the ability of our imaging system to reconstruct certain objects through a single scanning process. However, the highly specular nature of mmWave signals causes them to exhibit mirror-like reflections from targets, particularly those with flat surfaces, such as the metal target used in our experiments. Consequently, for large targets, not all reflections from the target propagate back to the mmWave receiver, and some parts of the target are absent in the image. To address this, we propose to reconstruct different parts of the target individually and then combine these images to recover the whole target. Specifically, we employ a multi-scanning strategy. Firstly, the user performs a zig-zag scanning pattern to ensure complete coverage of the imaging target shown in Fig. 7c. Next, the entire scanning trajectory is divided into several sub-trajectories based on the x-axis motion. For instance, the trajectory in Fig. 7d is segmented into four sub-trajectories, each moving along the x-axis for about 40 cm. For each sub-trajectory, we conduct imaging and estimate the phase error of the collected signals, which yields a partial image of the target, as shown in Fig. 7e. Finally, we stitch together the partial images to reconstruct the entire target, as depicted in Fig. 7f.

### Imaging without point references

**Fig. 8:**
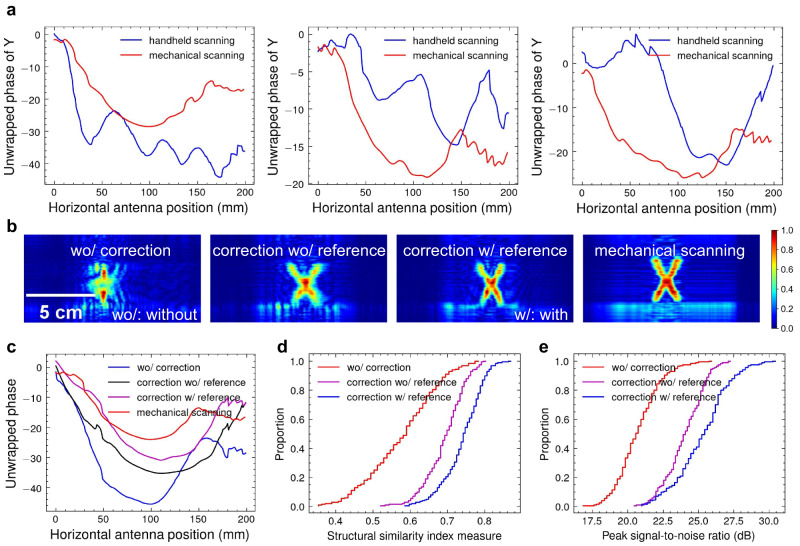
The performance of phase error estimation without the reference target. The experiments are conducted on the 200 handheld scanning samples illustrated in Fig. 6. **a** The phase history of non-point imaging targets (specifically, the letters Y, H, and M) that are obtained using mechanical scanning and handheld scanning. The overall trends in the phase history still exhibit quadratic-like behavior. **b** Qualitative comparison of handheld scanning images when performing no phase correction, phase correction without the reference target, and phase correction with the reference target. **c** Comparison of the phase history of the handheld scanning image in (**b**). It demonstrates that correcting the phase history without a reference target can reduce the phase error present in the original handheld scanning. **d** Empirical cumulative distribution functions in terms of peak signal-to-noise ratio and structural similarity index measure. Although the results may not be as optimal as with a reference target, phase correction without a reference target can still reduce phase errors and improve image quality compared to not applying any phase correction.

In this paper, we utilize a point target with a known ideal phase history (a quadratic curve) for phase error estimation. However, when such a point-like target is not present or cannot be observed by the radar due to occlusion, we can utilize other detectable targets, such as the imaging targets themselves, to estimate phase error. The key insight is that while the ideal phase histories of these non-point targets may not precisely follow a quadratic curve, their overall trend still exhibits quadratic-like behavior, as shown in Fig. 8a. Therefore, it is feasible to approximate these non-point targets as point targets and fit their actual phase history with a standard quadratic curve to obtain the phase errors (see the results of phase correction for multiple nearby targets in Supplementary Fig. 7). Although this approximation introduces estimation errors, these errors are significantly smaller compared to the phase errors caused by handheld scanning. Consequently, employing this phase error estimation approach still yields better imaging results compared to not employing any phase error estimation at all, as demonstrated in Fig. 8b, c.

## Discussion

In this study, we focus on overcoming the fundamental limitation of spatial resolution of mmWave devices by realizing handheld SAR imaging. Our proposed handheld system is capable of turning commercial-off-the-shelf mmWave radars into portable, affordable, and high-resolution imaging systems that can see through things, which has great potential in mobile applications, emergency situations, and limited-cost scenarios.

Estimating motion errors from raw radar signal, referred to as the autofocus technique^40^, is a well-studied field in airborne SAR^41,42^. Generally, typical autofocus methods can be classified into three categories: MapDrift^43^, phase gradient autofocus^44^, and image optimization approaches^45–48^. The MapDrift-based autofocus divides the whole aperture into two or more sub-apertures and estimates motion parameters by cross-correlating the resulting sub-images. The phase gradient autofocus-based approaches extract phase gradient from dominant scatters in the imaging scene and apply phase corrections that simultaneously sharpen all scatters. The image optimization autofocus approach searches for the optimal compensated phase to improve the image quality based on specific metrics. While these methods have been proven effective in space-borne and air-borne SAR, they cannot be directly applied to near-field 3D mmWave SAR systems for various reasons. First, the MapDrift-based autofocus can only cope with second-order phase error caused by incorrect speed estimation for linear motion. Second, the phase gradient autofocus-based techniques are based on a far-field assumption that all row pixels of SAR images are at an effectively equivalent range from the radar, which is not valid for near-field 3D SAR. Finally, for 3D SAR that has considerably more optimized variables, the image optimization approaches are also not suitable due to the increasing optimization difficulty and computational burden.

While the reconstruction performance of our system is promising, it does exhibit certain limitations. Specifically, residual phase errors stemming from rotational motion error and imprecise phase error estimation may still degrade the resulting images. To further refine the image quality, one can employ well-established image enhancement techniques, including learning-based^49,50^ and optimization-based approaches^51,52^, which have garnered advancements in the realm of image processing. Moreover, exceeding the maximum allowed motion errors may also lead to the failure of PSF optimization and image reconstruction. Despite this, we believe that this study represents a substantial stride forward in handheld 3D SAR imaging and will ignite further exploration within this promising field.

In our experiments, we anticipate users to move the radar in a near-straight line to emulate traditional strip-map SAR, which may not be a flexible operating pattern. In future work, we aim to explore techniques that enable handheld imaging with squiggle scanning paths, as well as investigate other SAR imaging techniques such as circular SAR or cylinder SAR. These advancements will further enhance the versatility and applicability of handheld mmWave SAR imaging.

In conclusion, this paper presented a practical handheld mmWave SAR imaging system. With the portability, affordability, and high-resolution 3D imaging ability, we believe that our design has the potential to become a standard component of subsequent handheld imaging systems, allowing more creative real-world applications in the future.

## Data avaliability

The data that support the findings of this study are available from the corresponding author upon reasonable request.

## Code avaliability

Code is available from the corresponding author upon reasonable request.

### Supplementary information

Supplementary information

## References

[1] Sheen D, McMakin D, Hall T (2001). Three-dimensional millimeter-wave imaging for concealed weapon detection. IEEE Trans. Microw. Theory Tech..

[2] Appleby R, Anderton RN (2007). Millimeter-wave and submillimeter-wave imaging for security and surveillance. Proc. IEEE.

[3] Zhang X (2020). Broadband millimeter-wave imaging radar-based 3-d holographic reconstruction for nondestructive testing. IEEE Trans. Microw. Theory Tech..

[4] Abou-Khousa MA, Rahman MSU, Donnell KM, Qaseer MTA (2023). Detection of surface cracks in metals using microwave and millimeter-wave nondestructive testing techniques—a review. IEEE Trans. Instrum. Meas..

[5] Taylor ZD (2015). Thz and mm-wave sensing of corneal tissue water content: In vivo sensing and imaging results. IEEE Trans. Terahertz Sci. Technol..

[6] Topfer F, Oberhammer J (2015). Millimeter-wave tissue diagnosis: the most promising fields for medical applications. IEEE Microw..

[7] Gao Y, Zoughi R (2017). Millimeter wave reflectometry and imaging for noninvasive diagnosis of skin burn injuries. IEEE Trans. Instrum. Meas..

[8] Hameed H (2022). Pushing the limits of remote RF sensing by reading lips under the face mask. Nat. Commun..

[9] Mercuri M (2019). Vital-sign monitoring and spatial tracking of multiple people using a contactless radar-based sensor. Nat. Electron..

[10] Mirbeik A, Ashinoff R, Jong T, Aued A, Tavassolian N (2022). Real-time high-resolution millimeter-wave imaging for in-vivo skin cancer diagnosis. Sci. Rep..

[11] Töpfer F, Dudorov S, Oberhammer J (2015). Millimeter-wave near-field probe designed for high-resolution skin cancer diagnosis. IEEE Trans. Microw. Theory Tech..

[12] Di Meo S (2017). On the feasibility of breast cancer imaging systems at millimeter-waves frequencies. IEEE Trans. Microw. Theory Tech..

[13] Bevacqua MT, Di Meo S, Crocco L, Isernia T, Pasian M (2021). Millimeter-waves breast cancer imaging via inverse scattering techniques. IEEE J. Electromagn. RF Microw. Med. Biol..

[14] Orr I (2021). Coherent, super-resolved radar beamforming using self-supervised learning. Sci. Robot..

[15] Wang S, Li S, An Q, Zhao G, Sun H (2022). Near-field millimeter-wave imaging via arrays in the shape of polyline. IEEE Trans. Instrum. Meas..

[16] Zhang W (2023). A grating-lobes suppression method for wideband mimo millimeter-wave imaging arrays. IEEE Trans. Antennas Propag..

[17] Li, S. et al. Millimeter-wave imaging via circular-arc mimo arrays. *IEEE Trans. Microw. Theory Tech*. 1–17 (2023).

[18] Tian X, Guo Q, Wang Z, Chang T, Cui H-L (2021). Pragmatic approach to phase self-calibration for planar array millimeter-wave mimo imaging. IEEE Trans. Instrum. Meas..

[19] Guo Q, Wang Z, Chang T, Cui H-L (2020). Millimeter-wave 3-d imaging testbed with mimo array. IEEE Trans. Microw. Theory Tech..

[20] Tian X, Wang Z, Chang T, Cui H-L (2022). Adaptive background clutter mitigation for millimeter wave mimo imaging. IEEE Trans. Geosci. Remote Sens..

[21] Batra A (2021). Short-range sar imaging from ghz to thz waves. IEEE J. Microwaves.

[22] I C-L, Han S, Bian S (2020). Energy-efficient 5g for a greener future. Nat. Electron..

[23] Surawy-Stepney T, Hogg AE, Cornford SL, Davison BJ (2023). Episodic dynamic change linked to damage on the thwaites glacier ice tongue. Nat. Geosci..

[24] Marom M, Goldstein RM, Thornton EB, Shemer L (1990). Remote sensing of ocean wave spectra by interferometric synthetic aperture radar. Nature.

[25] Holloway J, Wu Y, Sharma MK, Cossairt O, Veeraraghavan A (2017). Savi: Synthetic apertures for long-range, subdiffraction-limited visible imaging using fourier ptychography. Sci. Adv..

[26] Álvarez Narciandi G, López-Portugués M, Las-Heras F, Laviada J (2019). Freehand, agile, and high-resolution imaging with compact mm-wave radar. IEEE Access.

[27] Álvarez Narciandi G, Laviada J, Las-Heras F (2021). Freehand mm-wave imaging with a compact mimo radar. IEEE Trans. Antennas Propag..

[28] Álvarez Narciandi G, Laviada J, Las-Heras F (2021). Towards turning smartphones into mmwave scanners. IEEE Access.

[29] Schellberg, J. M., Regmi, H. & Sur, S. mmsight: towards robust millimeter-wave imaging on handheld devices. In *Proc. IEEE 24th International Symposium on a World of Wireless, Mobile and Multimedia Networks (WoWMoM)*, 117–126 (2023).

[30] Smith JW, Torlak M (2022). Efficient 3-d near-field mimo-sar imaging for irregular scanning geometries. IEEE Access.

[31] Saadat, M. S., Sur, S., Nelakuditi, S. & Ramanathan, P. Millicam: Hand-held millimeter-wave imaging. In *Proc. 29th International Conference on Computer Communications and Networks (ICCCN)*, 1–9 (2020).

[32] Regmi, H., Saadat, M. S., Sur, S. & Nelakuditi, S. Squigglemilli: approximating sar imaging on mobile millimeter-wave devices. *Proc. ACM Interact. Mob. Wearable Ubiquitous Technol*. **5**10.1145/3478113 (2021).

[33] Vasileiou, C. et al. Efficient cnn-based super resolution algorithms for MM wave mobile radar imaging. In *Proc. IEEE International Conference on Image Processing (ICIP)*, 3803–3807 (2022).

[34] Laviada J, Álvarez Narciandi G, Las-Heras F (2022). Artifact mitigation for high-resolution near-field sar images by means of conditional generative adversarial networks. IEEE Trans. Instrum. Meas..

[35] Wang Z, Guo Q, Tian X, Chang T, Cui H-L (2019). Near-field 3-d millimeter-wave imaging using mimo RMA with range compensation. IEEE Trans. Microw. Theory Tech..

[36] Lopez-Sanchez J, Fortuny-Guasch J (2000). 3-d radar imaging using range migration techniques. IEEE Trans. Antennas Propag..

[37] Gao Y, Ghasr MT, Zoughi R (2020). Effects of and compensation for translational position error in microwave synthetic aperture radar imaging systems. IEEE Trans. Instrum. Meas..

[38] Zhuge X, Yarovoy AG (2012). Three-dimensional near-field mimo array imaging using range migration techniques. IEEE Trans. Image Process..

[39] Yanik ME, Wang D, Torlak M (2020). Development and demonstration of mimo-sar mmwave imaging testbeds. IEEE Access.

[40] Chen J (2022). Motion compensation/autofocus in airborne synthetic aperture radar: a review. IEEE Geosci. Remote Sens. Mag..

[41] Tay C (2022). Sea-level rise from land subsidence in major coastal cities. Nat. Sustain..

[42] Engram M (2020). Remote sensing northern lake methane ebullition. Nat. Clim. Change.

[43] Calloway T, Donohoe G (1994). Subaperture autofocus for synthetic aperture radar. IEEE Trans. Aerosp. Electron. Syst..

[44] Wahl D, Eichel P, Ghiglia D, Jakowatz C (1994). Phase gradient autofocus-a robust tool for high resolution sar phase correction. IEEE Trans. Aerosp. Electron. Syst..

[45] Fienup JR (2000). Synthetic-aperture radar autofocus by maximizing sharpness. Opt. Lett..

[46] Morrison RL, Do MN, Munson DC (2007). Sar image autofocus by sharpness optimization: a theoretical study. IEEE Trans. Image Process..

[47] Wang J, Liu X (2006). Sar minimum-entropy autofocus using an adaptive-order polynomial model. IEEE Geosci. Remote Sens. Lett..

[48] Zeng T, Wang R, Li F (2013). Sar image autofocus utilizing minimum-entropy criterion. IEEE Geosci. Remote Sens. Lett..

[49] Zhang, K., Zuo, W., Gu, S. & Zhang, L. Learning deep cnn denoiser prior for image restoration. In *Proc. IEEE Conference on Computer Vision and Pattern Recognition (CVPR)* (2017).

[50] Zamir, S. W. et al. Multi-stage progressive image restoration. In *Proc. IEEE/CVF Conference on Computer Vision and Pattern Recognition (CVPR)*, 14821–14831 (2021).

[51] Figueiredo M, Nowak R (2003). An em algorithm for wavelet-based image restoration. IEEE Trans. Image Process..

[52] Zhang H, He W, Zhang L, Shen H, Yuan Q (2014). Hyperspectral image restoration using low-rank matrix recovery. IEEE Trans. Geosci. Remote Sens..

[53] Barket AR, Hu W, Wang B, Shahzad W, Malik JS (2022). Selection criteria of image reconstruction algorithms for terahertz short-range imaging applications. Opt. Express.

