# Supplementary material for: A high-resolution handheld millimeter-wave imaging system with phase error estimation and compensation
Source: Commun Eng. 2024 Jan 5;3:4. doi: 10.1038/s44172-023-00156-2 (PMC10956079; doi:10.1038/s44172-023-00156-2)
Supplement: Supplementary file 1 — Supplementary information [file 44172_2023_156_MOESM1_ESM.pdf]

Supplementary Information for "A High-resolution Handheld Millimetre-wave Imaging System with Phase Error Estimation and Compensation"

Yadong Li, Dongheng Zhang, Ruixu Geng, Zhi Lu, Zhi Wu, Yang Hu,
Qibin Sun, Yan Chen*
University of Science and Technology of China, Hefei, China.
Corresponding author: Yan Chen Email: eecyan@ustc.edu.cn

S.I. Supplementary Note 1

Experimental setup of mechanical scanning. The mechanical scanning is based on the TI MMWCAS-RF-EVM radar board working at 76-81 GHz, which can formulate an 86-element monostatic linear array that spans vertically along the y-axis. Then, the radar is horizontally moved along the x-axis using a motion controller to synthesize a 200 × 86 planar array. The spacing between adjacent antennas is 1 mm in the x-axis and 0.97 mm in the y-axis. This choice ensures accurate sampling and coverage of the imaging area.

S.II. Supplementary Note 2

Experimental setup of handheld scanning. During the handheld scanning, users hold the MIMO radar and horizontally move it along the x-axis for approximately 20 cm to 30 cm over a duration of 5 seconds. The radar's frame rate is set to 100 Hz, resulting in a total of 500 frames with each having 86 elements along the y-axis. Subsequently, we perform signal resampling to downsample the 500 frames to 200 frames, ensuring a uniform spacing of 1 mm along the x-axis. This allows us to obtain a synthetic planar array with a dimension of 200 $\times$ 86, which contains handheld phase errors along the z-axis. Finally, we conduct phase error estimation and imaging on these measurements to reconstruct the target.

S.III. Supplementary Note 3

Experimental setup of imaging and reference targets for different experiments.

Setup of Fig. 1. The imaging target, a metal letter A, is positioned at a distance of 300 mm from the array plane. The size of the target and the imaging area are 60 mm × 60 mm and 130 mm $\times$ 130 mm, respectively.

Setup of Fig. 3 and Fig. 5. The imaging target used is a corner reflector with an edge dimension of 5.5 cm. Its position relative to the center of the virtual array plane is at (0, 0, 0.65) m. Another corner reflector is used as the reference target with an edge dimension of 9.5 cm. It is positioned at (x_r, 0.3, 1.8) m, where $x_r \in$ [-0.2, 0.2] m. The x-axis distance between the imaging target and the reference target (i.e., x_r) ranges from -0.2 m to 0.2 m due to the limited field of view of the radar beam. Hence, the goal is to ensure that both targets can be detected by the radar while providing some flexibility on the x-axis position of the targets. The y-axis coordinate of the reference is 30 cm higher than the imaging target to avoid occlusion.

Setup of Fig. 6. The size of all metal letters is 6 cm × 6 cm. The reference target is a corner reflector with an edge dimension of 9.5 cm. The position of the imaging target relative to the center of the array plane is (0, 0, 0.3) m. The position of the reference target relative to the array plane is (x_r, 0.3, 1.5) m, where $x_r \in$ [-0.2, 0.2] m.

Setup of Fig. 7(a). The size of the knife is 23 cm × 3 cm. The reference target is a corner reflector with an edge dimension of 9.5 cm. The position of the imaging target relative to the center of the array plane is (0, 0, 0.3) m. The position of the reference target relative to the array plane is (x_r, 0.3, z_r) m, where $x_r \in$ [-0.2, 0.2] m and $z_r \in \{1, 2, 3\}$ m.

Setup of Fig. 7(b). The size of the scissor is 18 cm × 7 cm. The reference target is a corner reflector with an edge dimension of 9.5 cm. The position of the imaging target relative to the center of the array plane is (0, 0, 0.35) m. The position of the reference target relative to the array plane is (x_r, 0.3, 2) m, where $x_r \in$ [-0.2, 0.2] m.

Setup of Fig. 7(c). The size of the star-like object is 25 cm × 25 cm. The position of the imaging target relative to the center of the array plane is (0, 0, 0.55) m. To avoid the occlusion between the reference and the actual target, two corner reflectors are placed at (x_r, -0.3, 1.5) m and (x_r, 0.3, 1.8) m, where $x_r \in$ [-0.2, 0.2] m. The edge dimensions of the corner reflectors are 5.5 cm and 9.5 cm, respectively.

S.IV. Supplementary Note 4

To demonstrate the required phase correcting time for different numbers of acquisitions, we conduct the proposed phase error estimation and compensation on various numbers of acquisitions. Following the handheld scanning settings outlined in Supplementary Note 2, we can synthesize a virtual array of size 200 × 86. By increasing the scanning time and distance, we can obtain more acquisitions along the x-axis, ranging from 200 to 2000. The algorithms are executed 50 times for each number of acquisitions on a desktop equipped with an Intel Core i7-11700K CPU. The average computational time for each scenario is presented in Table I. As expected, the computational time increases with the number of acquisitions. However, it is worth noting that the proposed method demonstrates superior efficiency for long acquisitions (only 0.53 s for 2000 acquisitions). This is because the phase error estimation process does not require iterative optimization and can be effectively solved using the least square method. Hence, the computational time remains reasonable even for longer acquisitions, which is advantageous for practical implementation.

TABLE SI: Phase correcting time for different numbers of acquisitions.

Number of acquisitions	200	400	600	800	1000	1200	1400	1600	1800	2000
Time (s)	0.06	0.11	0.15	0.2	0.27	0.31	0.37	0.42	0.47	0.53

S.V. Supplementary Note 5

It is noteworthy that (i) we define **the ground truth of deviation from a regular acquisition** as the difference between an expected ideally linear trajectory (e.g., z = 0) and the ground truth of the non-linear handheld trajectory; (ii) the goal of computing the deviations is to assess the maximum handheld deviation that can be handled by our method. Obtaining the ground truth of the handheld trajectory, however, is challenging. Since the phase of mmWave signal is sensitive to sub-millimetre-level motion error, measuring the ground truth of the handheld trajectory requires costly motion capture systems that have sub-millimetre-level tracking accuracy. For instance, to achieve a 3D tracking accuracy of 0.1 mm, even the least expensive motion capture system made by OptiTrack costs more than $40,000 [*S1*]. Fortunately, we observe that the maximum deviation is usually at cm-level, as shown in Supplementary Fig. 2 and Supplementary Fig. 4, while the localization error of the tracking camera is at mm-level, as demonstrated in [*S2*]. Therefore, we use the tracking camera of the handheld imaging system to provide a coarse estimation of the maximum deviation. Specifically, we first use the tracking camera of the handheld imaging system to acquire the coarse handheld trajectory. Then we calculate **the estimated deviation from a regular acquisition** as the difference between the expected ideally linear trajectory (e.g., z = 0) and the coarse handheld trajectory given by the tracking camera. In summary, while we acknowledge that the tracking camera of our handheld system cannot provide the ground truth of the movement, we believe it is reasonable to use the tracking camera with a precision of mm-level to assess the maximum handheld deviations that are at cm-level.

S.VI. Supplementary Figure 1

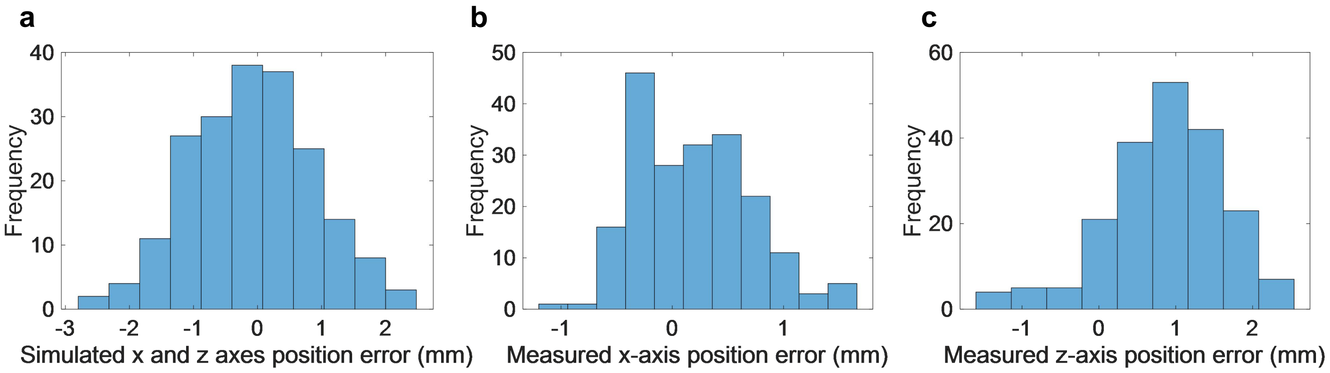

Supplementary Fig. 1. The histograms of the distribution of positioning errors in Fig. 1. (a) The simulated position error for Fig. 1(c) and (d), which follows a normal distribution with zero mean and 1 mm standard deviation. (b) and (c) are the measured position error for Fig. 1(e). They are obtained by co-locating the RealSense camera and the radar and then moving them with a motion controller.

S.VII. Supplementary Figure 2

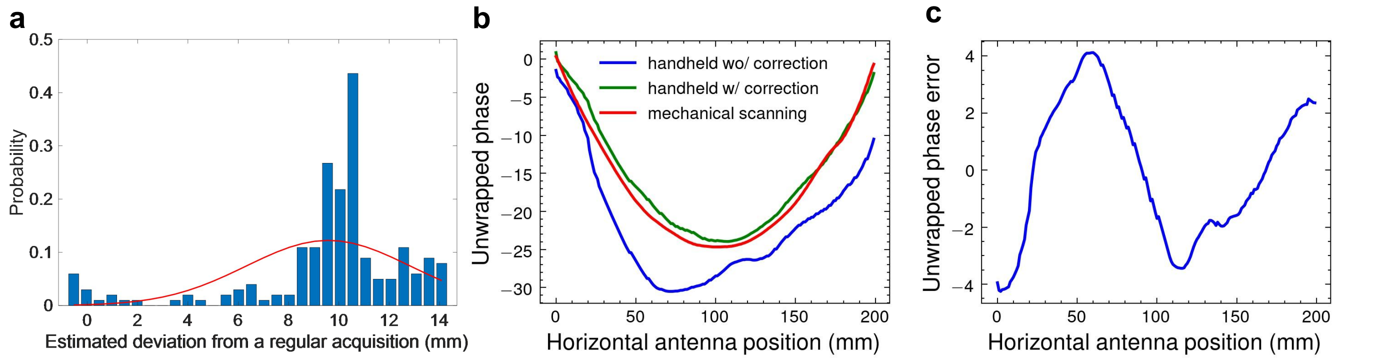

Supplementary Fig. 2. The estimated deviation from a regular acquisition and the phase error of the imaging target in Fig. 3. (a) The histogram of estimated deviation from a regular acquisition by measuring the difference between an expected ideally linear trajectory (e.g., $z = 0$) and that given by the tracking camera of the handheld system. (b) The phase history of handheld scanning after phase correction is depicted, and it demonstrates consistency with the phase history obtained from mechanical scanning, which indicates that the phase correction effectively mitigates the phase errors introduced during handheld scanning. (c) The estimated phase error of the imaging target is obtained by computing the difference between the phase history acquired through handheld scanning and the estimated quadratic phase history.

S.VIII. Supplementary Figure 3

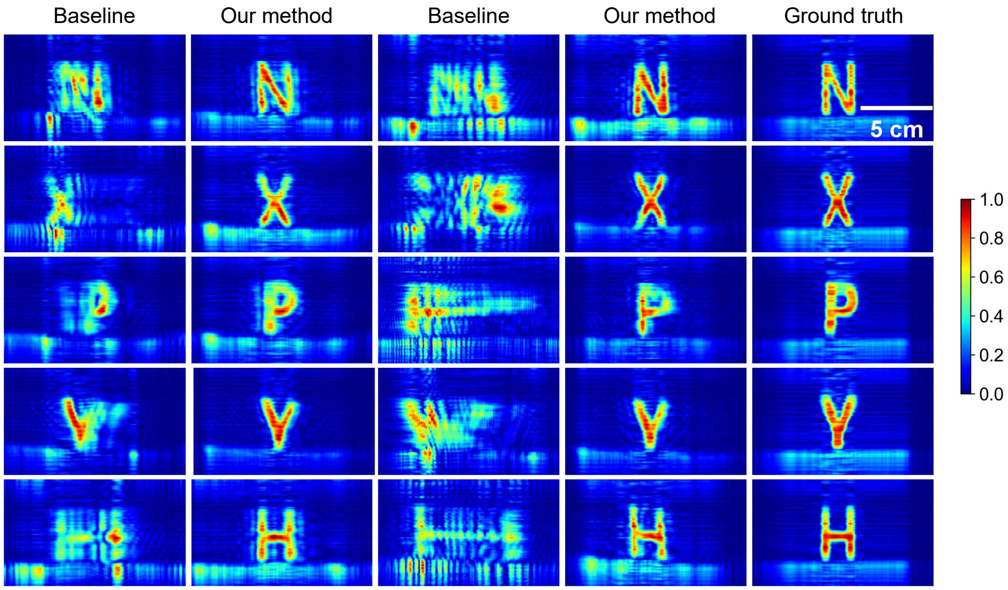

Supplementary Fig. 3. Qualitative results of another 5 letters. Our proposed approach can effectively reconstruct targets with different shapes and reduce the defocus effect.

S.IX. Supplementary Figure 4

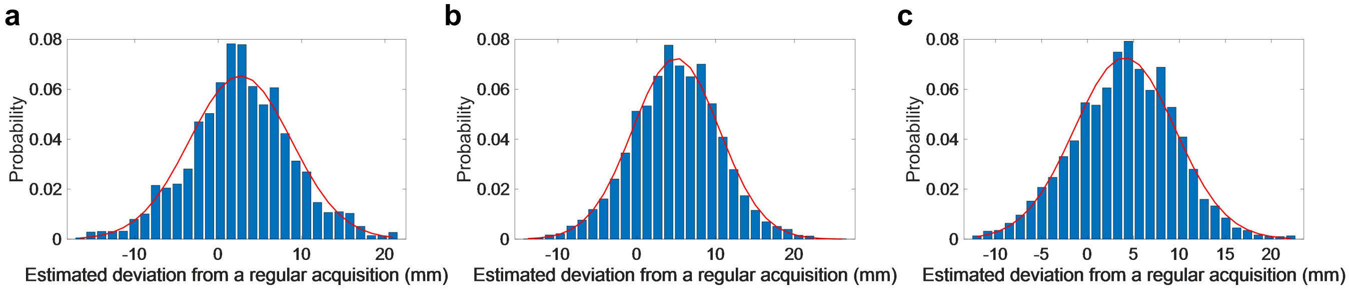

Supplementary Fig. 4. The histogram of estimated deviation from a regular acquisition by measuring the difference between an expected ideally linear trajectory (e.g., z = 0) and that given by the tracking camera of the handheld system. (a) The estimated deviation from a regular acquisition of 100 handheld scanning samples in Fig. 5. (b) The estimated deviation from a regular acquisition of 200 handheld scanning samples in Fig. 6. (c) The estimated deviation from a regular acquisition of handheld scanning samples whose structural similarity index measure is above 0.75 after phase correction in Fig. 6, which approximately reflects the motion error that our approach can handle.

S.X. Supplementary Figure 5

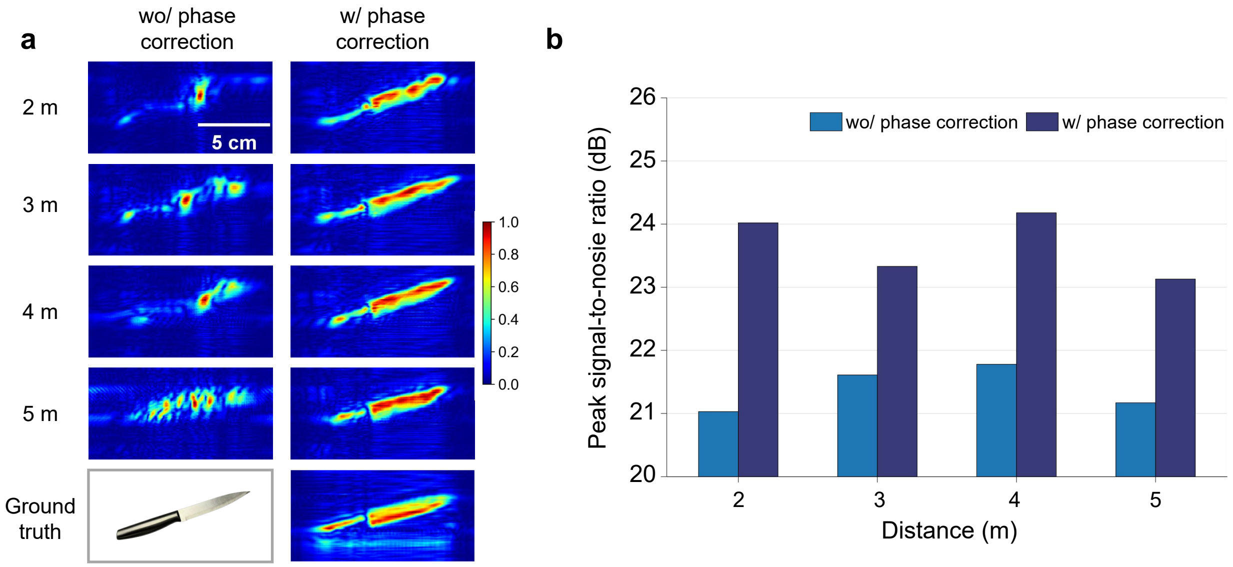

Supplementary Fig. 5. Performance when the reference target is placed at different distances. We collect handheld scanning samples with the reference target placed 2 m, 3 m, 4 m, and 5 m away from the radar. Each scenario has 35 samples, with a total of 140 samples for 4 different distances. The imaging target is a knife that is 0.3 m away from the radar. (a) Qualitative results of phase correction for different distances. (b) Quantitative analysis of phase correction for different distances. The imaging results and peak signal-to-noise ratio after phase correction show that our approach is not impacted by the distance of the reference target.

S.XI. Supplementary Figure 6

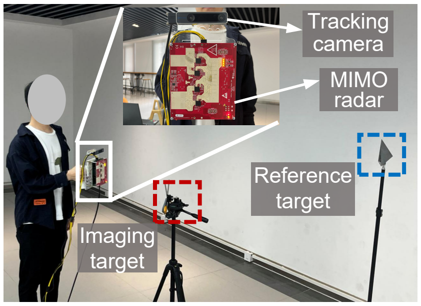

Supplementary Fig. 6. Experimental setup. The imaging system consists of a tracking camera and a multi-input-multi-output (MIMO) radar. The imaging scene includes the imaging target and the reference target.

S.XII. Supplementary Figure 7

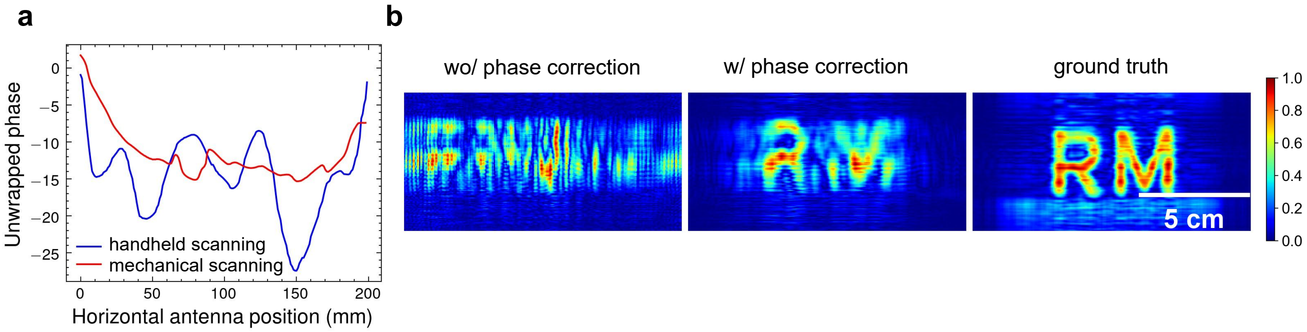

Supplementary Fig. 7. Phase correction for multiple targets in the same range bin. (a) The phase history of letters R and M of mechanical and handheld scanning. (b) The performance of phase correction. If the distance between the targets is smaller than the range resolution of the radar system, the phase history will be the combination of these targets, deviating more from the quadratic curve. However, our proposed phase correction approach can still provide some level of correction in this scenario.

S.XIII. Supplementary references

References

[S1] "Optitrack." https://www.optitrack.com/systems/#robotics/primex-41/6.

[S2] G. Álvarez Narciandi, J. Laviada, and F. Las-Heras, "Towards turning smartphones into mmwave scanners," *IEEE Access*, vol. 9, pp. 45147–45154, 2021.
